# Content Quality of YouTube Videos About Gestational Diabetes: Systematic Evaluation

**DOI:** 10.2196/30156

**Published:** 2022-04-07

**Authors:** Eleanor M Birch, Karolina Leziak, Jenise Jackson, Emma Dahl, Charlotte M Niznik, Lynn M Yee

**Affiliations:** 1 Division of Maternal-Fetal Medicine Department of Obstetrics & Gynecology Feinberg School of Medicine, Northwestern University Chicago, IL United States

**Keywords:** diabetes, gestational diabetes, health information, internet, web-based, pregnancy, YouTube

## Abstract

**Background:**

People with gestational diabetes have enhanced learning requirements during pregnancy, and management of their disease often requires the translation of health information into new health behavior changes. Seeking information from the internet to augment learning from health professionals is becoming more common during pregnancy. YouTube is a popular free and accessible web-based resource, which may be particularly useful for individuals with low health literacy or other barriers to receiving high-quality health care; however, the quality and content of YouTube videos varies, and little is known about those covering gestational diabetes.

**Objective:**

We aimed to systematically evaluate the quality, content, and reliability of YouTube videos about gestational diabetes.

**Methods:**

A systematic search of YouTube videos was conducted over the course of 1 week in April 2020 using the following keywords: “gestational diabetes,” “gestational diabetes management,” “gestational diabetes treatment,” and “pregnancy and diabetes.” The search results were displayed by relevance, replicating a default YouTube search attempt. The first 60 results from each keyword were reviewed (n=240). Exclusion criteria were videos unrelated to gestational diabetes, videos not in English, and those for which the full video was not available at the time of review. For each unique video, a gestational diabetes content score was used to rate video comprehensiveness and accuracy, and the DISCERN instrument, a validated metric to assess consumer health information, was used to evaluate the reliability of information presented. Videos were further categorized by quality: videos with DISCERN scores lower than 3 (out of 5) or a content score less than 4 (out of 7) were categorized as low quality, and all others were designated high quality. We performed descriptive analysis and compared video characteristics by source and quality rating.

**Results:**

For 115 unique videos, the mean content score (out of 7) was 3.5 (SD 2.0) , and the mean DISCERN score (out of 5) was 2.7 (SD 0.7), representing low to moderate information comprehensiveness and reliability respectively. Video sources were categorized as personal vlog (12/115, 10.4%), web-based education (37/115, 32.2%), medical (52/115, 45.2%), business or company (13/115, 11.3%), and media clip (1/115, 0.9%). DISCERN and content scores trended higher among medical and web-based education videos. The majority of videos (n=88) were categorized as low quality, while 27 videos were categorized as high quality. Video duration was longer for high-quality videos (*P*<.001); high- and low-quality videos otherwise had similar views and viewer interaction numbers.

**Conclusions:**

Although high-quality videos about gestational diabetes exist, reliability, accuracy, and comprehensiveness were low overall, and higher quality was not associated with increased viewer interaction. It is important to acknowledge the limitations of this platform and to assist patients in accessing high quality content and differentiating the quality of information sources.

## Introduction

Gestational diabetes is one of the most common complications of pregnancy. Untreated or undertreated gestational diabetes is associated with perinatal and maternal complications, including preeclampsia, macrosomia, neonatal hypoglycemia, and maternal risk of developing type 2 diabetes [1]. Because management of gestational diabetes may include a combination of nutritional therapy, physical activity, blood glucose monitoring, or medication, affected individuals require health behavior changes beyond those required during routine pregnancy care in order to have best outcomes [2-4]. These increased requirements are particularly noteworthy because individuals with gestational diabetes are more likely to belong to racial, ethnic, and socioeconomic groups that face barriers to access to high-quality care [1,5,6]. Because an individual’s access to and application of informative resources about gestational diabetes testing, treatment, and self-management may determine their risk of adverse pregnancy outcomes, improving access to high-quality health information and support for health behavior change is crucial [6-10].

The internet is a widely available, increasingly accessed resource for health information, and it is a common source of information for pregnancy-related health concerns [11,12]. Multiple studies [13-17] have found high prevalences of internet and other digital media use among pregnant patients in the United States—estimated to be greater than 70% among patients of different ages, races or ethnicities, and socioeconomic groups. Web-based information-seeking has been reported for many pregnancy-related topics including fetal development, pregnancy complications, prenatal care, medication safety, nutrition, and gestational weight gain [15,17-19]. The rapidity of information access and availability of supplemental explanation are reported advantages of seeking information from the internet [17]. YouTube is a particularly important source of health information, both because of its popularity as one of the most used web-based platforms, and because its information is unregulated [20-25]. Evaluations on a variety of topics, including infertility treatment, medication safety during pregnancy, and diabetes outside of pregnancy have reported variability in the value of YouTube as a health information source and some found that videos provided incomplete or misleading information, while others have reported advantages, including the availability of personal experience–based information [26-28]. However, little is known about the content and quality of information from YouTube on gestational diabetes.

Given the emerging role of web-based platforms—including YouTube—as health information resources, and the importance of optimizing health care information and interventions for individuals with gestational diabetes, it is important to learn more about the content and quality of resources about gestational diabetes that are available on YouTube. Restrictions on access to care [8], limited health literacy [18], and lack of availability of culturally appropriate interventions [8,29,30] are well-known barriers in the provision of prenatal care for gestational diabetes, and understanding resources—such as videos available on YouTube—that may help combat those barriers is vital. Thus, our objective was to examine the quality and content of videos about gestational diabetes in order to ultimately develop opportunities for better, more accessible health care delivery.

## Methods

### Search Strategy

We systematically reviewed YouTube content on gestational diabetes on April 30, 2020. Search terms were identified in consultation with 2 perinatal diabetes care specialists (CN and LY), and Google Trends was used to identify top searches associated with gestational diabetes. The search was then conducted on a cache-cleared web browser using incognito mode with the following terms: “gestational diabetes,” “gestational diabetes management,” “gestational diabetes treatment,” and “pregnancy and diabetes.” The search was conducted via public access—a registered account was not used.

Search results were displayed by relevance, which is the default search setting. The first 60 results—representing the first 3 pages of results—from each keyword search were reviewed and analyzed. The sample size and sort strategy were selected based on research indicating that the majority of internet searchers view only 1 page of results, and 83% of searchers view no more than 3 pages [31]. Duplicate videos, videos unrelated to gestational diabetes, videos in a language other than English, and clips for which the full video was unavailable were excluded.

### Data Extraction

Descriptive characteristics of each video were collected, including video length, number of comments, channel number of subscribers, number of views, number of likes, and number of dislikes. Collection of this information was completed by 1 reviewer (EB), over the course of 1 week (from April 26, 2020 to May 2, 2020) to minimize variability in number of views and other characteristics collected. Source characteristics were also gathered from each video. These included the video source and whether the video was a *character video*, which was defined as a video in which a specific, identifiable person was presenting. Video source was determined based on the affiliation of each video’s author when available (eg, identified as an employee of a hospital system or pharmaceutical company), or the channel description and theme (eg, medical school test preparation videos or personal vlog in which the author presents primarily personal experiences).

### Content and Quality Assessment

Criteria to judge video content were formulated from American College of Obstetricians and Gynecologists recommendations for management of gestational diabetes, and in consultation with perinatal diabetes care specialists (CN and LY). Video comprehensiveness and accuracy of information provided about (1) screening or testing guidelines; (2) treatment for gestational diabetes self-management; (3) nutrition information; (4) information about the purpose of gestational diabetes treatment and benefits; (5) blood glucose level monitoring guidelines; (6) gestational diabetes effects on long-term maternal health; and (7) gestational diabetes effects on the fetus. These topics were selected because they represent key foundational knowledge regarding gestational diabetes that is essential for participants to understand their condition, participate in self-monitoring and management, and engage in health care to prevent adverse perinatal outcomes. To evaluate videos, we used a content score—1 point for each content area addressed accurately in the video (ie, the video included relevant information in that topic area, though depth of explanation varied); thus, a maximum score of 7 was possible if a video covered the comprehensive range of topics about gestational diabetes accurately. Similar assessments have been performed in prior evaluations of YouTube videos as a health information source [32-36].

The DISCERN instrument [37] was used to assess the quality and reliability of the videos as information sources. The DISCERN instrument consists of 16 questions assessing the (1) reliability, (2) quality, and (3) overall rating of web-based publications. DISCERN criteria are written as questions and are rated on a scale from 1 (not satisfied) to 5 (fully satisfied). The DISCERN tool has been validated for use in a variety of settings, and it has been used in similar studies that have evaluated YouTube videos as a source of health information [34,35,38-42].

A combination of both scoring techniques was used to divide videos into high- and low-quality categories. Videos with a mean DISCERN score less than 3 or a content score less than 4 were defined as low quality; otherwise, videos were defined as high quality. These quality cutoffs were chosen because they correspond to a DISCERN rating representing potentially important shortcomings or an inclusion of fewer than half of the expert-developed content topics, respectively, both of which affect the overall quality of the video as a reliable comprehensive source of information.

After determining evaluation criteria, 2 videos were assessed collaboratively by 3 reviewers (EB, JJ, ED) to establish consensus on the application of these criteria and scoring. Subsequently, these 3 reviewers independently evaluated the same 15 randomly selected videos for content and reliability. The remaining videos were then divided evenly among the reviewers, and each was evaluated using the established standardized criteria. Data for each video were collected and stored using REDCap (Vanderbilt University) software.

### Statistical Analysis

Statistical analyses were performed using Excel software (version 2020; Microsoft Inc). Interrater agreement was analyzed using intraclass correlation coefficients and single-factor analysis of variance. Descriptive statistics were used to evaluate study data. Frequencies were used to describe categorical variables; normally distributed continuous variables were presented as means, and nonnormally distributed variables as medians. The Kruskal-Wallis test for intergroup comparison, Spearman rank correlation, and Wilcoxon rank-sum test were used for analysis involving nonnormally distributed continuous variables. A *P* value<.05 was considered significant.

### Ethics Statement

This study was deemed exempt from review by the Northwestern University institutional review board because it does not involve human participants.

## Results

**Figure 1:**
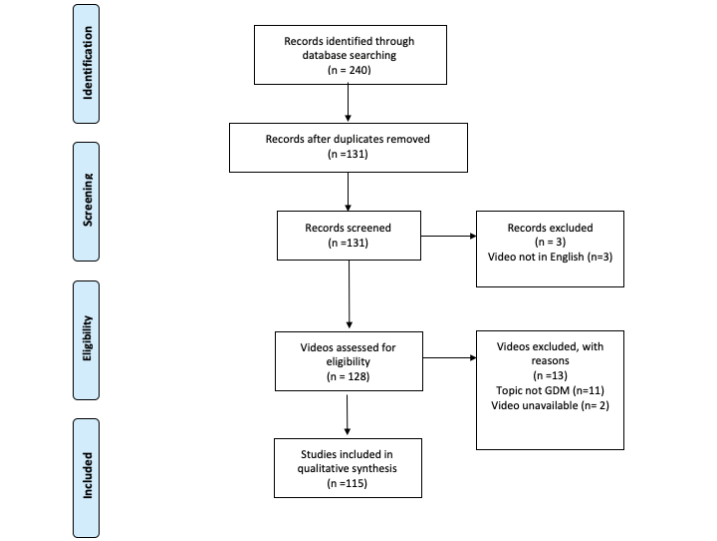
PRISMA (Preferred Reporting Items for Systematic Reviews and Meta-analyses) flow diagram. GDM: gestational diabetes mellitus.

The mean scoring disparity for the 15 videos was 0.27 (for DISCERN), and correlation was 88% (intraclass correlation 0.81), indicating a high level of interrater agreement. Of the 240 videos initially identified, 109 were duplicates, 3 were in a language other than English (Urdu, Nepali, Bengali), 2 with full videos unavailable at the time of data recording, and 11 had a primary topic that was not gestational diabetes. After applying exclusion criteria, 115 videos remained for analysis (Figure 1; Multimedia Appendix 1). Video characteristics (Table 1) varied widely, for example, the number of comments ranged from 0 to 651, and the number of channel subscribers ranged from 0 to 2 million. Out of 115 videos, 64 videos were categorized as character videos.

**Figure 2:**
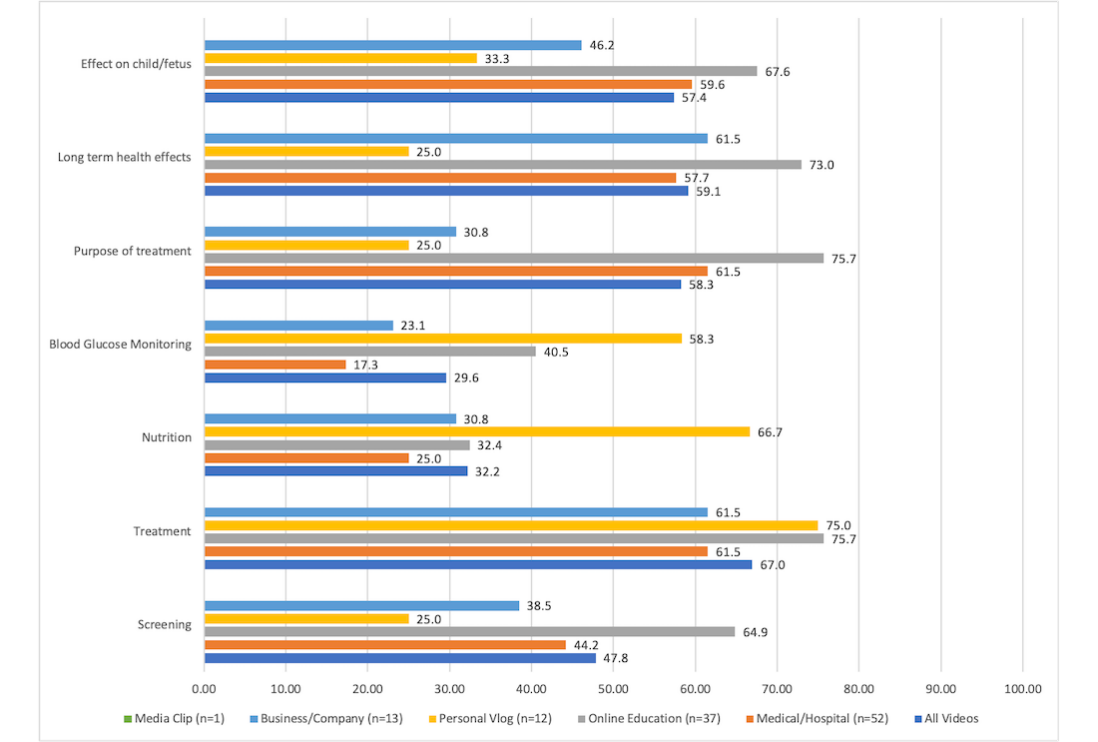
YouTube video content inclusion by topic area.

DISCERN scores (Table 2) ranged from 1.4 to 5, but were, on average, low (mean 2.70, SD 0.73). Content scores were similarly low; on average, videos contained accurate information on 3.5 out of the 7 topics (SD 2.01). The majority of videos (77/115, 67%) contained information about some type of gestational diabetes treatment, including medical nutritional therapy, physical activity, insulin, or oral medications. However, fewer videos contained information about nutritional guidelines for patients with gestational diabetes or about blood glucose monitoring (Figure 2). Of all video characteristics, only duration was significantly positively associated with both DISCERN score (*P*<.001) and topic score (*P*<.001).

The sources of videos included medical institutions or hospitals (52/115, 45.2%), web-based education (37/115, 32.2%), personal vlog (12/115, 10.4%), business or company (13/115, 11.3%), and media (1/115, 0.9%). The single video in the media category had the second highest number of views, comments, and subscribers; the highest number of dislikes; and was in the top 10 videos by number of likes; however, because the media category had only 1 video, it was not included in comparisons of characteristics by source. Among other sources (Table 3), personal vlogs were longer (*P*<.001), with more comments (*P*<.001), likes (*P*=.001), and dislikes (*P*=.01); there was no significant difference in views (*P*=.22) or subscribers (*P*=.60). The mean DISCERN score was highest for web-based education videos (mean 2.91, SD 0.79), followed by that for videos from medical institutions or hospital (mean 2.69, SD 0.64). Web-based education was the source with the highest average topic score (mean 4.3, SD 1.93).

Of 115 videos reviewed, 27 videos were designated as high quality, and 88 videos were designated as low quality. The low-quality group included 12 videos that were rated as low only because they included fewer than 4 topics; their DISCERN scores ranged from 3.0 to 3.6. Therefore, the scoring system did not result in a large number of videos with reliable in-depth information about a small number of topics, but instead, a small number of videos with moderate reliability and low topic comprehensiveness, being found in the low-quality categorization. Longer video duration was associated with higher quality (*P*<.001), but the numbers of views, comments, likes, dislikes, and subscribers were not associated with quality (Table 4). Among high-quality videos, 24 out of 27 were from web-based education or medical sources, 2 were personal vlogs, and 1 was from a business or company.

**Table 1:** Characteristics of the videos (n=115).

Video characteristic	Median (IQR)	Minimum to maximum
Duration (seconds)	234 (114-598)	34-3722
Views	4045 (808-23,809)	57-548,409
Comments	1 (0-8)	0-651
Likes	21 (3-112)	0-5700
Dislikes	1 (0-7)	0-1600
Channel subscribers	10,300 (750-60,000)	0-2,700,000

**Table 2:** Reliability and content score of YouTube videos about gestational diabetes by source.

Source		DISCERN			Content	
		Score, mean (SD)	*P* value	Score, mean (SD)		*P* value
**Total**		2.70 (0.73)	.05	3.51 (2.01)		.05
	Medical institution or hospital (n=52)	2.69 (0.64)	—^a^	3.27 (1.77)		—
	Web-based education (n=37)	2.91 (0.79)	—	4.30 (1.93)		—
	Personal vlog (n=12)	2.54 (0.56)	—	3.08 (2.18)		—
	Business or company (n=13)	2.38 (0.80)	—	2.92 (2.20)		—
	Media clip (n=1)	1.40 (—)	—	0 (—)		—

^a^No data or not applicable.

**Table 3:** Characteristics of YouTube videos about gestational diabetes by source.

Source	Characteristic, median (IQR)					
	Duration	Views	Comments	Likes	Dislikes	Subscribers
Medical (n=52)	192 (112-270)	2943 (612-14,576)	0 (0-3)	10 (2-45)	0 (0-4)	14,150 (2232-58,175)
Web-based education (n=37)	337 (164-975)	4045 (820-25,896)	2 (0-10)	19 (4-157)	1 (0-10)	6250 (268-79,900)
Personal vlog (n=12)	677 (438-974)	10,206 (5186-54902)	27 (4-143)	254 (48-699)	10 (2-29)	18,950 (1835-77,475)
Business or company (n=13)	113 (70-309)	1388 (702-23,902)	0 (0-4)	6 (2-47)	2 (0-6)	6090 (156-40,700)
Media clip (n=1)	58 (—^a^)	409,285 (—)	389 (—)	834 (—)	1600 (—)	2,100,000 (—)

**Table 4:** Characteristics of YouTube videos about gestational diabetes by quality.

Characteristic	Low quality (n=88), median (IQR)	High quality (n=27) ), median (IQR)	*P* value
Views	3851.5 (762.5-23,439.2)	4523 (1253- 24,533)	.63
Comments	1 (0-8.8)	0 (0-4)	.40
Likes	21 (2.3-114.3)	19 (4-84)	.90
Dislikes	1 (0-7.8)	2 (0-4)	.86
Duration	203.5 (98-374)	760 (263-1320)	<.001
Subscribers	13,950 (695-62,925)	6430 (819-38,300)	.90

## Discussion

### Principal Findings

In this study, we identified no relationship between video quality and indicators of video popularity. These findings indicate that the information available to pregnant individuals with gestational diabetes is highly variable in quality, and may not deliver accurate or comprehensive information to users.

### Comparison With Prior Work

YouTube has been evaluated as a source of health information on a variety of topics, including emerging infectious diseases and pregnancy [43,44], medication use in pregnancy [27], and diabetes outside of pregnancy [34], as well as colorectal cancer [36], ankylosing spondylitis [39], and rheumatoid arthritis [40]. The average DISCERN score in this study is concordant with that found in many previous studies of YouTube videos [34,35,38-40,43]. Scores in previous studies have typically fallen in the low to moderate reliability range, indicating potentially important shortcomings. Although studies evaluating other health conditions used various topic-specific comprehensiveness scales, they found similar levels of topic coverage, accuracy, and comprehensiveness to those found by us [36,40,41]. We found that this level of topic inclusion represents notably incomplete coverage of the health condition. The majority of videos we evaluated covered only 3 to 4 key topics about gestational diabetes accurately, and only 1 topic—gestational diabetes management—was included by more than 50% of videos. The overall low comprehensiveness of individual videos evaluated has implications for their utility as health information resource. In further research, it could be helpful to investigate YouTube user viewing patterns to ascertain whether these shortcomings can be overcome through careful combination of videos and topics covered.

Interestingly, in our study, one of the lowest rated videos overall by both DISCERN criteria and topic coverage had some of the highest views, likes, comments, and channel subscribers. This video was a media clip related to a television personality; therefore, the high numbers of views, likes, comments, and subscribers are likely because viewers accessed the clip for entertainment rather than for information about gestational diabetes. However, this highlights an important aspect of finding health information on YouTube—the search algorithm may not take the quality of the video into account for any specific search term, and quality varies significantly between videos. As we found and as has been seen previously, there are rare videos available with both good reliability and comprehensive topic coverage; however, it is difficult to ensure that these videos are accessed, as they are not necessarily the most viewed, liked, or highly ranked videos available [38,45,46].

### Clinical and Research Implications

Pregnant patients increasingly seek health education via web-based sources, including YouTube, and web-based media can be helpful adjuncts to clinical care [15,17,25,47-49]. Trust in information found on the internet is often reported to be high, and many people who sought information on the internet did not discuss it with their providers [15,17]. Therefore, it is important for providers to be aware of the overall use and limitations of YouTube as a resource when counselling patients with gestational diabetes, and it may be useful for clinicians to query and understand the resources patients are using to augment their clinical care.

However, it is also important to note that YouTube has the potential to address the specific needs of individuals with gestational diabetes. Health information and supportive social networks—which are both easily accessed via the internet—have been found to influence the challenges experienced by people with gestational diabetes [49-51]. In addition, patients’ health literacy levels and access to information are known to affect glycemic control and gestational diabetes–related outcomes [52-55]; interventions for pregnant individuals at an appropriately targeted health literacy level are particularly essential to address health inequities present in gestational diabetes care and outcomes [8,56]. Video-based information has frequently been used to combat care barriers related to health literacy [57-59]. Despite the limitations found in this study, YouTube videos remain a low-cost, easily accessible resource, and further interventions should investigate ways to address these limitations in order to harness YouTube’s potential as a patient education tool. Clinicians also have the opportunity to assist patients who are using YouTube as resource by recommending specific videos, discussing ways to identify reliable resources, or even creating high-quality content. Health care providers involved in gestational diabetes care are likely to have both the knowledge base and the resources available to create accurate comprehensive videos with information that is helpful for patients with gestational diabetes.

### Limitations

To the best of our knowledge, this study is the first to assess the reliability, content, and quality of YouTube videos about gestational diabetes, but it has several limitations. First, while the sample size was chosen to encompass the majority of videos likely to be encountered by individuals searching for information about gestational diabetes, we cannot ensure that all videos that a user may find were included, given the dynamic nature of video uploading. Search history or location may also affect ranking and accessibility of videos in ways that were not demonstrated in this paper. Second, we could not evaluate the target audience for each video, because most videos do not indicate their audience and default search setting will not necessarily preclude people from accessing videos aimed at different groups. As such, our assessment of quality and reliability may not reflect all videos with which pregnant individuals engage. Additionally, our search was limited to English-language videos, and future work is necessary to corroborate our findings with those for videos in other languages.

We also noted little gender, racial, or ethnic diversity among YouTube presenters in videos about gestational diabetes. Especially because social media sites, and YouTube specifically, may be sought as a more personal or personal-experience based resource, this lack of diverse representation is a noteworthy limitation. Based on the representation found in the videos evaluated, many patients may not see themselves or their experiences reflected fully.

### Conclusions

Despite the relatively low quality and comprehensiveness found among YouTube videos for individuals with gestational diabetes, YouTube is an easily accessible and increasingly important source of health information. Providers caring for individuals with gestational diabetes should remain aware of the limitations of this resource, especially when discussing information sources with patients. Further study may be helpful in elucidating ways to harness the potential strengths of YouTube for providing high-quality accessible health information.

## Appendix

Multimedia Appendix 1 List of videos (n=115) included in study.
